# Biofilm characterisation of *Mycoplasma bovis* co-cultured with *Trueperella pyogenes*

**DOI:** 10.1186/s13567-025-01468-1

**Published:** 2025-01-30

**Authors:** Koji Nishi, Satoshi Gondaira, Yuki Hirano, Masahide Ohashi, Ayano Sato, Kazuya Matsuda, Tomohito Iwasaki, Takuya Kanda, Ryoko Uemura, Hidetoshi Higuchi

**Affiliations:** 1 Animal Health Unit, Department of Veterinary Science, School of Veterinary Medicine, Rakuno Gakuen University, Ebetsu, Hokkaido Japan; 2 Monbetsu Veterinary Clinic, Hokkaido Agricultural Mutual Aid Association, Monbetsu, Hokkaido Japan; 3 Animal Research Center, Agricultural Research Department, Hokkaido Research Organization, Shintoku, Hokkaido Japan; 4 Large Animal Clinical Science, Department of Veterinary Science, School of Veterinary Medicine, Rakuno Gakuen University, Ebetsu, Hokkaido Japan; 5 Department of Veterinary Pathology, Department of Veterinary Science, School of Veterinary Medicine, Rakuno Gakuen University, Ebetsu, Hokkaido 069-8501 Japan; 6 Department of Food Science and Human Wellness, College of Agriculture, Food and Environment Science, Rakuno Gakuen University, Ebetsu, Hokkaido Japan; 7 Food Safety Laboratory, Faculty of Veterinary Medicine, Okayama University of Science, Imabari, Ehime Japan; 8 Animal Health, Faculty of Agriculture, University of Miyazaki, Miyazaki, Japan

**Keywords:** Antibiotics, antimicrobial agents, bovine respiratory disease, extracellular matrix, trachea

## Abstract

*Mycoplasma* pneumonia, caused by *Mycoplasma bovis* (*Mycoplasmopsis bovis*; *M. bovis*), is linked with severe inflammatory reactions in the lungs and can be challenging to treat with antibiotics. Biofilms play a significant role in bacterial persistence and contribute to the development of chronic lesions. A recent study has shown that polymicrobial interactions between species are an important factor in biofilm formation, yet the precise mechanism of biofilm formation in *M. bovis* remains unknown. By assuming multiple pathogen infections in the bovine respiratory disease complex (BRDC), this study examined the characterisation of the polymicrobial relationship between *M. bovis* and *Trueperella pyogenes* (*T. pyogenes*) during biofilm formation. Autopsies were performed on four Holstein calves (two chronic *Mycoplasma* pneumonia calves and two control calves). Bacterium-like aggregation structures (> 10 μm), which were assumed to be biofilms of *M. bovis* in vivo, were observed adhering to the cilia in calves with *Mycoplasma* pneumonia. *M. bovis* released an extracellular matrix to connect with neighbouring bacteria and form a mature biofilm on the plate. Biofilm formation in the co-culture of *M. bovis* and *T. pyogenes* (strain T1: 1 × 10^5^ and 1 × 10^6^ CFU/well) significantly increased (*p* < 0.05 and *p* < 0.01; 64.1% and 64.8% increase) compared to that in a single culture of these bacteria. Furthermore, some large aggregates (> 40 μm), composed of *M. bovis* and *T. pyogenes*, were observed. The morphological characteristics of this biofilm were similar to those observed in vivo compared to a single culture. In conclusion, the polymicrobial interaction between *M. bovis* and *T. pyogenes* induces biofilm formation, which is associated with increased resistance to antimicrobial agents, and this exacerbates the progression of chronic *Mycoplasma* pneumonia.

**Supplementary Information:**

The online version contains supplementary material available at 10.1186/s13567-025-01468-1.

## Introduction

*Mycoplasma bovis* (*Mycoplasmopsis bovis*; *M. bovis*) is a pathogen that causes mastitis [1], pneumonia [2], arthritis [3], and otitis media [4] in dairy cattle. *Mycoplasma* pneumonia caused by *M. bovis* is linked with severe inflammatory responses in the lungs. Treating conditions caused by this pathogen with antibiotics can be difficult and may result in substantial economic losses for dairy and beef farms [5]. Additionally, *M. bovis* is a central etiological agent of the bovine respiratory disease complex (BRDC). BRDC is caused by an interaction between viral and bacterial pathogens such as bovine herpesvirus, bovine viral diarrhoea virus, *Pasteurella multocida*, *Mannheimia haemolytica*, and *Trueperella pyogenes* (*T. pyogenes*) [6]. Each pathogen possesses unique features, such as suppressing the protective barrier function of the respiratory epithelium or the immune response in leukocytes [6, 7], which can result in chronic pneumonia. *T. pyogenes* is an opportunistic pathogen that forms part of the biota of the skin and mucous membranes of the upper respiratory, gastrointestinal, and urogenital tracts of animals [8]. The pathogen is involved in polymicrobial diseases, including mastitis, uterine infections, and pneumonia [8]. Consequently, *T. pyogenes* is often isolated from mixed infections of various bacterial species.

The expression levels of virulence genes, including *plo, fimA, nanH,* and *cbpA*, in *T. pyogenes* isolates in co-culture with *Fusobacterium necrophorum* (*F. necrophorum*) and *Escherichia coli* (*E. coli*) have been shown to increase [9]. This finding demonstrates that polymicrobial infection intensifies and exacerbates the diseases associated with *T. pyogenes* infection*.* Furthermore, a previous study reported that *M. bovis* and *T. pyogenes* can be isolated from lesions of chronic caseous pneumonia in cattle [10]. Therefore, it is suggested that co-infection with *M. bovis* and *T. pyogenes* can induce a more severe inflammatory reaction in respiratory tissues than a single infection. However, the detailed mechanism behind this inflammatory reaction remains unknown.

Biofilms, which are communities of microorganisms attached to biotic or abiotic surfaces [11], play a significant role in the persistence of bacteria and contribute to chronic lesions. Moreover, antibiotic and immune responses struggle to reach the bacteria within the biofilms [12]. The structure of the extracellular polymeric substance (EPS) matrix of the biofilm is composed of extracellular polysaccharides, DNA, and proteins [11]. A recent study showed that EPS material, produced and shared by multiple pathogens in co-culture, facilitates interspecies interactions by establishing compact microcolony structures during biofilm formation [13]. For example, the interaction between *Staphylococcus aureus* (*S. aureus*) and *Candida albicans* (*C. albicans*) demonstrates synergistic activity, significantly enhancing biofilm formation and contributing to an increase in antimicrobial resistance in *S. aureus* [14]. Thus, the polymicrobial interaction between species is significant in forming biofilm.

Moreover, *M. bovis* is known to form biofilms despite possessing a limited number of genes [15]. A previous study showed that the morphological characterisation of *M. bovis* biofilm on the plate and its formation potential may be associated with the expression of an adhesion factor [15]. However, these biofilms have never been observed in vivo, and the effect of polymicrobial relationships on biofilm formation remains undetermined. Therefore, in this study, we analysed the morphological characteristics of *M. bovis* biofilm related to spontaneous *Mycoplasma* pneumonia in calves. Additionally, we examined the characterisation of the polymicrobial relationship between *M. bovis* and *T. pyogenes* during biofilm formation to clarify its relevance to the development of pneumonia.

## Materials and methods

### Animals

Two 2-month-old Holstein calves (Calf 1 and Calf 2) with chronic *Mycoplasma* pneumonia and two control Holstein calves without clinical respiratory symptoms, aged 1 and 2 months, underwent autopsies immediately after euthanasia. Calf 1 and Calf 2 also had arthritis and showed lameness. The autopsies were conducted at Rakuno Gakuen University in 2021 (Hokkaido, Japan), following the Guide for the Care and Use of Laboratory Animals of the School of Veterinary Medicine.

Tracheal samples and caseous necrotic foci from the lung were analysed by polymerase chain reaction (PCR) using *M. bovis*-specific primers, as previously described [16]. Swabs from these samples were cultured on blood agar plates (Eiken Kagaku, Tokyo, Japan) and incubated for 48 h at 37 °C. The bacterial colony obtained was identified by its 16S ribosomal RNA gene sequence, as previously described [17].

The sequence of the 16S 27F forward primer was 5′-AGAGTTTGATCCTGGCTCAG-3′, and the sequence of the 1492R reverse primer was 5′-GGTTACCTTGTTACGACTT-3′. PCR products were extracted using the FastGene Gel/PCR Extraction Kit (Nippon Genetics, Tokyo, Japan). The product sequence was analysed by Hokkaido System Science Co., Ltd. The sequence data was confirmed through database analysis using the Basic Local Alignment Search Tool (BLAST).

### Bacterial strains

Table 1 lists the seventeen *M. bovis* strains and two *T. pyogenes* strains employed in this study. Before use, the *M. bovis* and *T. pyogenes* strains were cultured in a modified pleuropneumonia-like organisms (PPLO) medium (Kanto Kagaku, Tokyo, Japan) and brain heart infusion supplemented with 5% foetal bovine serum (FBS). Both strains were subsequently stored at −80 °C.

**Table 1:** **Information regarding the bacterial strains**

Species	Strain	Origin	Disease
*M. bovis*	PG45	ATCC 25523	–
	Strain M1	Nasal cavity	Pneumonia
	Strain M2	Nasal cavity	Pneumonia
	Strain M3	Nasal cavity	Pneumonia
	Strain M4	Nasal cavity	Pneumonia
	Strain M5	Nasal cavity	Pneumonia
	Strain M6	Nasal cavity	Pneumonia
	Strain M7	Synovial fluids	Arthritis
	Strain M8	Synovial fluids	Arthritis
	Strain M9	Milk	Mastitis
	Strain M10	Milk	Mastitis
	Strain M11	Lung	Pneumonia
	Strain M12	Heart	Endocarditis
	Strain M13	Heart	Endocarditis
	Strain M14	Heart	Endocarditis
	Strain M15	Heart	Endocarditis
	Strain M16	Lung (*Mycoplasma* pneumonia Calf1)	Pneumonia
*T. pyogenes*	Strain T1	Lung (*Mycoplasma* pneumonia Calf1)	Pneumonia
	Strain T2	Lung (*Mycoplasma* pneumonia Calf2)	Pneumonia

### Light microscopy and immunohistochemical analysis

The tracheal tissues were fixed in a 4% paraformaldehyde solution, dehydrated through an ethanol gradient from 70 to 100%, and embedded in paraffin. Following deparaffinisation using xylene, the tissue sections were stained with haematoxylin and eosin (H&E) and observed under a light microscope. Indirect immunofluorescence analysis was used for immunohistochemical staining. The sections were heated in a microwave oven in the presence of a 0.01 M sodium citrate buffer (pH 6.0) for 15 min and then immersed in a 3% hydrogen peroxide solution at room temperature for 10 min.

After pretreatment, the sections were incubated and blocked using 5% normal goat serum for 20 min at room temperature. The sections were then incubated with rabbit anti-cytokeratin18 (Proteintech, Chicago, IL, USA) and mouse anti-*M. bovis* (Millipore, Billerica, MA, USA) antibodies at room temperature for 2 h. This step was followed by incubation with rhodamine-conjugated goat anti-rabbit IgG antibody (Thermo Fisher Scientific, Waltham, MA, USA) and Alexa Fluor 488-conjugated goat anti-mouse IgG antibody (Thermo Fisher Scientific) at room temperature for 1 h. The sections were stained with a DAPI (4′,6-diamidino-2-phenylindole) solution (DOJINDO, Kumamoto, Japan) at room temperature for 10 min and visualised using a Nikon C2 laser confocal microscope (Nikon, Tokyo, Japan).

The images obtained were analysed using NIS Elements Advanced Research (AR) Analysis and Fiji (a distribution of ImageJ software from the US National Institutes of Health, Bethesda, Maryland, USA) using the following methods [18].

### Biofilm formation of *M. bovis* single culture

Biofilm formation was performed with modifications as previously described [15]. Five strains of *M. bovis* (PG45, strains M1–M4) were cultured in a modified PPLO medium at 37 °C for 24 h without aeration. After culturing, 10 μL of planktonic *M. bovis* was inoculated, in triplicate, into a non-coated 96-well cell culture plate (NIPPON Genetics: flat-bottom).

Ten different culture media were used for biofilm formation. These included modified PPLO medium (PPLO rich), unmodified PPLO broth medium (not adding horse serum and yeast extract, etc. PPLO broth: Kanto Kagaku), Mueller–Hinton (MH: Beckton Dickinson, Franklin Lakes, NJ, USA), lysogeny broth (LB: Beckton Dickinson), brain heart infusion (BHI: Beckton Dickinson), trypticase soy broth (TSB: Beckton Dickinson), Todd-Hewitt broth (THB: Kanto Kagaku), Dulbecco’s modified Eagle’s medium [DMEM: Fujifilm Wako (Osaka, Japan)], DMEM supplemented with 5% FBS, and Roswell Park Memorial Institute 1640 medium (RPMI: Fujifilm Wako), and RPMI supplemented with 5% FBS to determine the appropriate medium.

These media were added at 190 μL per well in a 96-well plate. The planktonic *M. bovis* culture was then diluted to 1:20 and incubated at 37 °C with 5% CO_2_ for 24 h without aeration to preserve biofilm.

### Biofilm formation in co-culture with *M. bovis *and *T. pyogenes*

The stored *M. bovis* was cultured in a modified PPLO medium at 37 °C for 24 h, and 10 μL of the cultured planktonic *M. bovis* was subsequently inoculated into a non-coated 96-well plate (NIPPON Genetics: flat-bottom). The stored *T. pyogenes* was centrifuged (7000 rpm, 5 min, 4 °C), suspended in a PPLO broth medium and then inoculated at concentrations of 1 × 10^4^, 10^5^, 10^6^, and 10^7^ colony-forming units (CFU) per 10 μL into a 96-well plate. PPLO broth medium was added at 180 μL per well in a 96-well plate and incubated at 37 °C with 5% CO_2_ for 24 h without aeration to preserve biofilm.

### Crystal violet staining

The biofilm in the 96-well plate was washed thrice with phosphate-buffered saline (PBS) to remove planktonic cells and then fixed with 99.5% methanol for 15 min. The biofilm was then stained with a 2% crystal violet solution for 20 min and rinsed thrice with distilled water. The plate was dried and decolourised with 200 μL of 99.5% ethanol to release the crystal violet, which was quantified using a microplate reader (Bio-Rad, Hercules, CA, USA) by measuring the absorbance at 595 nm.

### Scanning electron microscope

The cultured planktonic *M. bovis* was prepared as described above and inoculated with 300 μL into a 35 mm non-coated single culture dish (IWAKI, Chiba, Japan) containing 2700 μL of PPLO broth medium. *T. pyogenes* was inoculated at 3 × 10^8^ CFU/well and incubated at 37 °C with 5% CO_2_ for 24 h without aeration to preserve biofilm. Cultured bacterial biofilms and tracheal tissues isolated from pneumonia-affected and control calves were fixed with half-strength Karnovsky’s solution at 4 °C overnight.

The tissue samples were post-fixed with 1% osmium tetroxide for 30 min. The tissues and biofilm samples were then washed with 0.1 M cacodylate buffer and dehydrated using an ethanol gradient ranging from 30 to 100% (with concentrations at 30%, 50%, 70%, 80%, 90%, 95%, and 100%), with 10 min spent at each step. Following the t-butyl alcohol freeze-drying method, the specimens were coated with Pt–Pd and observed at 8 kV under a HITACHI S-2460N electron microscope.

### Confocal microscope

The cultured planktonic *M. bovis* was prepared as described and inoculated with 60 μL into a 24-well non-coated culture dish (IWAKI, Chiba, Japan) containing a 12 mm round cover glass and 540 μL of PPLO broth medium. The *T. pyogenes* was inoculated at 6 × 10^7^ CFU/well and incubated at 37 °C with 5% CO_2_ for 24 h without aeration to preserve biofilm.

The bacterial biofilm on the round cover glass was stained with DAPI solution (DOJINDO) or the LIVE/DEAD® BacLight™ Bacterial Viability Kit (Thermo Fisher Scientific) and washed thrice with PBS. The samples were fixed with a 4% paraformaldehyde solution and observed using a Nikon C2 laser confocal microscope. The images obtained were analysed using NIS Elements AR Analysis and Fiji.

### Statistical analysis

The optical density (OD) values of biofilm biomass are shown as mean ± the standard error of triplicate well data. The Shapiro–Wilk test was performed to assess the normality of biofilm biomass data, with the results indicating a normal distribution for all groups, allowing parametric testing. The data were compared using a two-tailed analysis of variance (ANOVA) followed by Dunnett’s test for significant differences using the statistical analysis program EZR [19]; Saitama Medical Center, Jichi Medical University, Saitama, Japan]. A probability (*p*) value < 0.05 was considered to indicate a statistically significant difference in all cases.

## Results

### Pathological findings and morphological analysis of *Mycoplasma pneumonia* in calves

**Figure 1:**
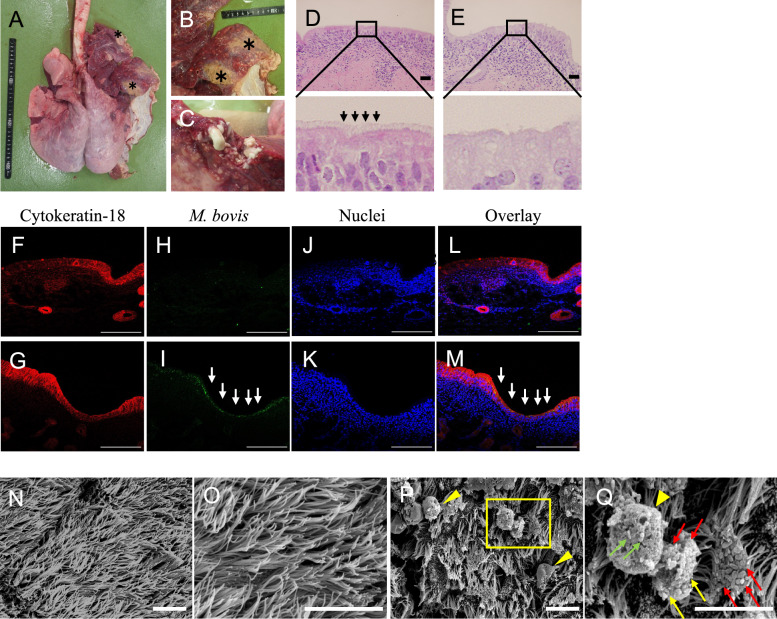
**Pathological findings and morphological analysis of *Mycoplasma* pneumonia in calves.** **A** and **B** Calf infected with *M. bovis* and *T. pyogenes* showed caseous necrotic foci in the right cranial part and middle lobe (asterisk). **C** Caseous discharge was observed on the cut surface of the right cranial part. **D** and **E** Tracheal tissues from calves with no clinical respiratory symptoms (controls) and those with *Mycoplasma* pneumonia were stained with haematoxylin and eosin. Cilia were observed on the epithelial cells of the control calves (block arrows). Representative images from two experiments are shown. Scale bar: 32 μm. **F**–**M** Tracheal tissues from control calves and those with *Mycoplasma* pneumonia were stained with cytokeratin-18 antibody, *M. bovis* antibody, and DAPI solution and analysed using a fluorescence microscope. Cytokeratin-18 is stained red (**F** and **G**), *M. bovis* is stained green (**H** and **I**), and the nucleus is stained blue (**J** and **K**). These images are overlayed (**L** and **M**). Representative images from two experiments are shown. Areas positive for *M. bovis* were detected in the epithelial cells of tracheal tissues from calves with *Mycoplasma* pneumonia (white arrows). Scale bar: 100 μm. **N**–**Q** The tracheal mucosa from control calves and those with *Mycoplasma* pneumonia were analysed by SEM. Representative images are shown. **N** and **O**) The tracheal mucosa surface of the control calves was completely covered with cilia. Scale bar: 10 μm. **P** Bacterium-like aggregation structures were observed on the tracheal mucosa from calves with *Mycoplasma* pneumonia (yellow arrowheads). Scale bar: 10 μm. The yellow square was magnified in (**Q**) to provide a better view. **Q** Bacteria were detected on the cilia (red arrows). The boundary between bacterial cells was obscured (yellow arrows). Bacteria were detected on the bacterium-like aggregation structures (green arrows). Scale bar: 5 μm.

Two control calves with no clinical respiratory symptoms and two calves affected by *Mycoplasma* pneumonia were subjected to pathological autopsies to evaluate the formation of *M. bovis* biofilm in vivo. The calf with *Mycoplasma* pneumonia (Calf 1) showed caseous necrotic foci in the right cranial part and middle lobe (Figure 1A and B). The cut surface of the right cranial region displayed evidence of caseous discharge (Figure 1C).

Tracheal tissues were obtained from control calves without clinical respiratory symptoms and those with *Mycoplasma* pneumonia. PCR testing revealed the presence of *M. bovis* and *T. pyogenes* in the tracheal mucosa swabs and caseous necrotic foci in the lungs of the calves with *Mycoplasma*. Tracheal tissues were stained with H&E (Figures 1D and E). Cilia were aligned on the epithelial cells of the tracheal mucosa in control calves but not in calves with *Mycoplasma* pneumonia.

Tissues from the tracheas of control calves and those with *Mycoplasma* pneumonia were stained with cytokeratin-18 antibody, *M. bovis* antibody, and DAPI solution. The tissues were then analysed using a fluorescence microscope (Figures 1F–M). Areas positive for *M. bovis* were identified in the epithelial cells of the tracheal tissues of calves with *Mycoplasma* pneumonia (Figures 1I and M). The micromorphological characterisation of the tracheal mucosa in control calves and those with *Mycoplasma* pneumonia was analysed using structural equation modelling (SEM) (Figures 1N–Q). Notably, the surface of the tracheal mucosa in the control calves was completely covered with cilia (Figures 1N and O).

Furthermore, the clumping of cilia on the tracheal mucosa of calves with *Mycoplasma* pneumonia decreased compared to the control calves (Figure 1P). Bacterium-like aggregation structures (> 10 μm) were observed adhering to the cilia in calves with *Mycoplasma* pneumonia (Figures 1P and Q, yellow arrowheads). Additionally, bacteria that appeared 0.4–0.5 μm pleomorphic and coccoid were detected on the cilia of the tracheal mucosa (Figure 1Q, indicated by red arrows). The boundaries between bacteria were also obscured, and bacterial aggregation structures were observed (Figure 1Q, indicated by green arrows). These characteristic structures were also observed in other control and *Mycoplasma* pneumonia calves (Additional file 1). *Mycoplasma* pneumonia Calf 2 showed caseous necrotic foci in the right and left cranial part lobe.

PCR detected *M. bovis* and *T. pyogenes* in the tracheal mucosa swabs and caseous necrotic foci in the lungs of *Mycoplasma* pneumonia Calf 2. *M. bovis* antigen was detected in the epithelial cells and submucosal gland in the tracheal tissues of *Mycoplasma* pneumonia Calf 2 by fluorescence microscope. Additionally, the surface of the tracheal mucosa of control calves was completely covered with cilia. In contrast, *Mycoplasma* pneumonia in calves showed loss of cilia on the tracheal mucosa. Furthermore, bacterium-like aggregation structures (> 10 μm) were observed in calves with *Mycoplasma* pneumonia.

### Quantitative analysis of *M. bovis* biofilm formation

**Figure 2:**
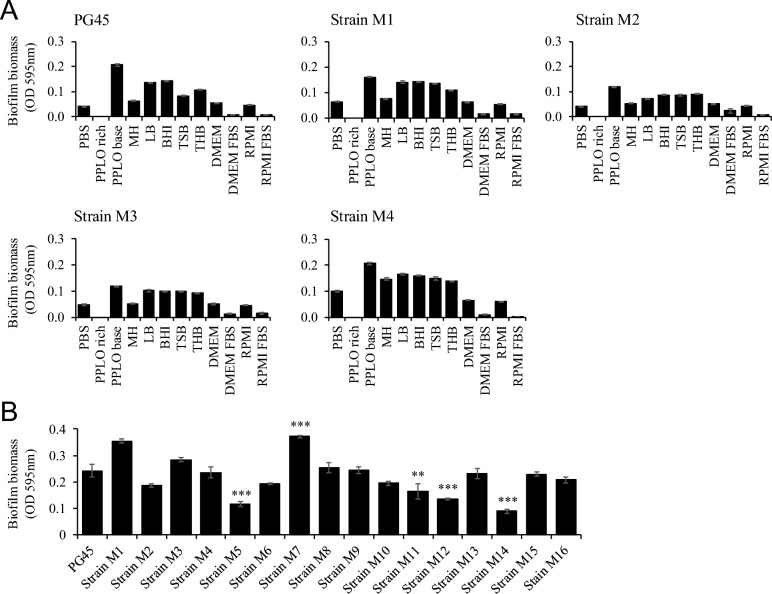
**Quantitative analysis of biofilm formation of *M. bovis.*** **A** Five strains of *M. bovis* (PG45, strains M1–M4) were cultured in eleven culture media on a 96-well microplate (flat-bottom). *M. bovis* biofilm was quantified using crystal violet staining. *PBS* phosphate buffer saline, *PPLO rich* modified PPLO medium, *PPLO broth* unmodified PPLO broth medium, *MH* Mueller–Hinton, *LB* lysogeny broth, *BHI* brain heart infusion, *TSB* trypticase soy broth, *THB* Todd–Hewitt broth, *DMEM* Dulbecco’s modified Eagle’s medium, *DMEM + FBS* 5% foetal bovine serum in DMEM, *RPMI* Roswell Park Memorial Institute 1640 medium, *RPMI + FBS* 5% foetal bovine serum in RPMI. **B** Biofilm formation of seventeen *M. bovis* strains was quantified using crystal violet staining in PPLO broth medium for 24 h. The OD values of biofilm biomass are shown as mean ± the standard error of representative in three independent experiments with triplicate well. Significant difference to PG45 (**: *p* < 0.01; ***:* p* < 0.001).

We first determined which culture medium was most appropriate to evaluate the biofilm formation of *M. bovis*. Next, five strains of *M. bovis* were cultured in 96-well microplates across eleven culture media for 24 h before being stained with crystal violet (Figure 2A). Notably, biofilm from the five *M. bovis* strains was not detected in the modified PPLO medium (PPLO rich, with horse serum and yeast extract), which is a major growth medium for mycoplasmas. In contrast, cultures in the unmodified PPLO broth medium (without horse serum or yeast extract) showed the highest level of biofilm formation among the eleven culture media. Furthermore, the biofilm formation of five strains of *M. bovis* in DMEM or RPMI was higher than that in DMEM + FBS or RPMI + FBS, respectively. Therefore, in subsequent experiments, an unmodified PPLO broth medium was used to evaluate the biofilm formation of *M. bovis*.

Seventeen *M. bovis* strains were cultured in PPLO broth medium and stained with crystal violet (Figure 2B). The *M. bovis* strains M1 and M7 were found to be strong producers of biofilm (OD value > 0.3). On the other hand, strains M5, M11, M12, and M14 were shown to be weak producers of biofilm (OD value < 0.2). PG45 was identified as a moderate biofilm producer (OD value was 0.26 ± 0.02). Additionally, a significant difference was observed compared to strains M5 (*p* < 0.001), M7 (*p* < 0.001), M11 (*p* < 0.001), M12 (*p* < 0.01), and M14 (*p* < 0.001).

Therefore, the results show that the ability to form biofilms differs depending on the strains.

### Morphological characterisation of *M. bovis* biofilm in single culture

**Figure 3:**
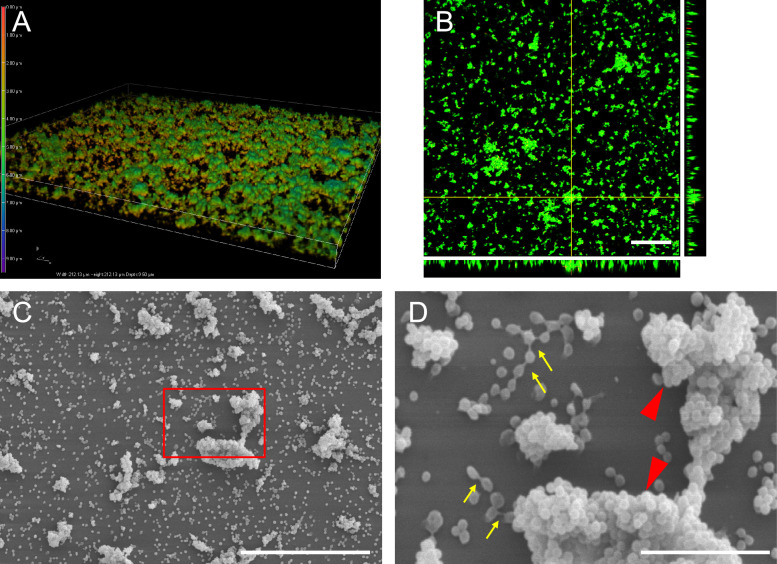
**Morphological characterisation of *M. bovis* biofilm.** **A** Topographical image of *M. bovis* biofilm was analysed using a confocal microscope. *M. bovis* (strain M7) was cultured in PPLO broth for 24 h and then stained with DAPI solution. A representative image from three independent experiments is shown. **B** Orthogonal sections showing horizontal (z) and side views (x and y) of three-dimensional biofilm images reconstructed by confocal microscopy. *M. bovis* (strain M7) was stained with LIVE/DEAD BacLight, resulting in live bacteria appearing green and dead bacteria appearing red. A representative image from three independent experiments is shown. Scale bar: 50 μm. **C** *M. bovis* biofilm (strain M7) was analysed by SEM. Scale bar: 20 μm. The red square was magnified in (**D**) to provide a better view. **D** *M. bovis* was connected by filamentous structures and arranged in a rosary-like formation (yellow arrows). *M. bovis* aggregation was observed (red arrowheads). These assays were performed in triplicates in three independent experiments. A representative image from three independent experiments is shown. Scale bar: 5 μm.

Strain M7, a strong biofilm producer, was stained with DAPI solution and observed using a confocal microscope (Figure 3A). *M. bovis* was observed as aggregations of various sizes and scattered formations. *M. bovis* was stained with LIVE/DEAD BacLight and visualised in orthogonal sections (Figure 3B).

The *M. bovis* aggregate was constructed with live cells, with almost no dead cells. The micromorphology of *M. bovis* was evaluated by SEM (Figures 3C and D). A single bacterium adhering to the plate and approximately 10 μm bacterial aggregates were observed (Figure 3D, red arrowheads). *M. bovis* cells were connected by filamentous structures and arranged in a rosary-like formation (Figure 3D, indicated by yellow arrows). Aggregation and concatenated bacterial structures were observed in these strains, which showed moderate levels of biofilm formation, lower than that in strain M7 (Additional file 2).

### Quantitative analysis of biofilm formation in co-culture with *M. bovis* and *T. pyogenes*

**Figure 4:**
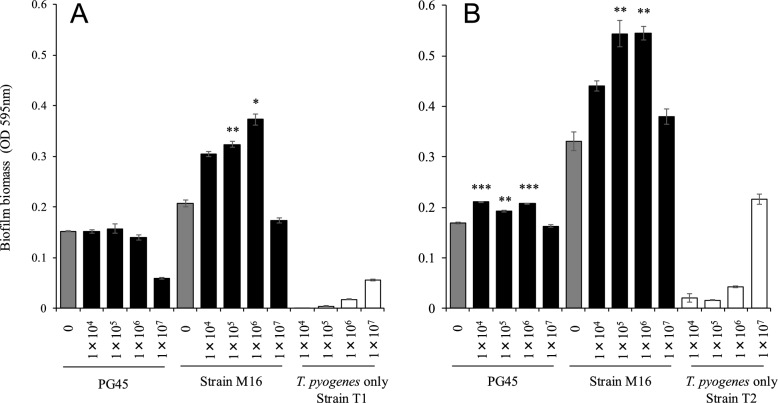
**Quantitative analysis of biofilm formation in co-culture with *M. bovis* and *T. pyogenes.**** M. bovis* strains PG45 and M16 were co-cultured with 1 × 10^4^, 1 × 10^5^, 1 × 10^6^, or 1 × 10^7^ CFU of *T. pyogenes* (**A**: strain T1, **B**: strain T2) per well in PPLO broth medium for 24 h. Biofilms were quantified using crystal violet staining. Grey bars represent *M. bovis* in a single culture; black bars represent *M. bovis* and *T. pyogenes* in co-culture; white bars represent *T. pyogenes* in a single culture. The OD values of biofilm biomass are shown as mean ± the standard error of triplicate well. Experiments using strain T1 (**A**) and strain T2 (**B**) were performed in three and one experiment, respectively. Significant difference to each *M. bovis* in single culture (*: *p* < 0.05; **: *p* < 0.01; ***:* p* < 0.001).

*M. bovis* strains PG45 or M16 (isolated from Calf 1) and *T. pyogenes* (strain T1 isolated from Calf 1; strain T2 isolated from Calf 2) were co-cultured in a 96-well microplate using a PPLO-based medium for 24 h and then stained with crystal violet (Figure 4). Biofilm biomass of strain M16, when co-cultured with 1 × 10^5^ or 1 × 10^6^ CFU/well of *T. pyogenes* (strain T1 and T2), were significantly higher (*p* < 0.01 and < 0.05) than those of the single culture.

The biofilm biomass of PG45, when co-cultured with 1 × 10^4^, 1 × 10^5^, or 1 × 10^6^ CFU/well of *T. pyogenes* (strain T2), were also significantly higher (*p* < 0.01 and < 0.001) than those of the single culture. *T. pyogenes* in single culture were shown to be a weak biofilm producer (OD value < 0.1). However, the biofilm formation of PG45 and strain M16, when co-cultured with 1 × 10^7^ CFU of *T. pyogenes,* was lower than when co-cultured with 1 × 10^4^, 1 × 10^5^, or 1 × 10^6^ CFU/well of *T. pyogenes*.

### Morphological characterisation of biofilm in co-culture with *M. bovis* and *T. pyogenes*

**Figure 5:**
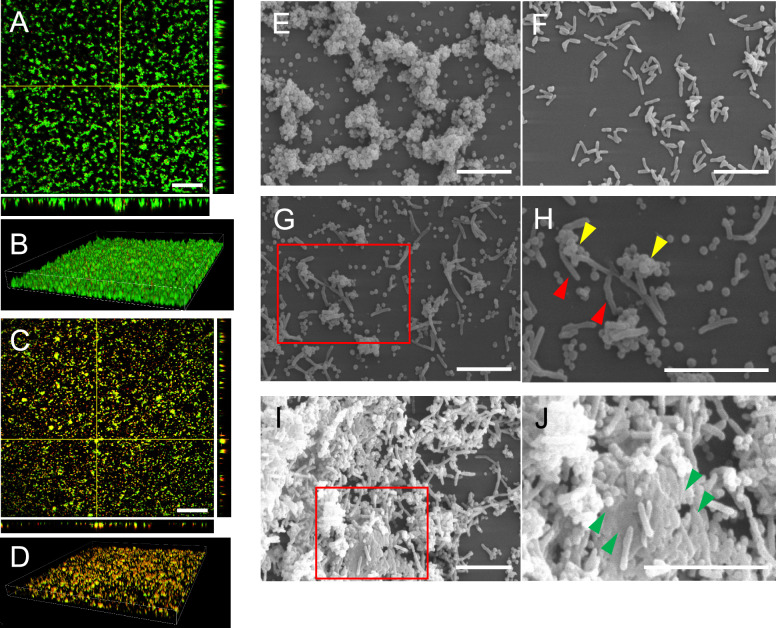
**Morphological characterisation of biofilm co-cultured with *M. bovis* and *T. pyogenes.*** **A** and **B** *M. bovis* (strain M16) was cultured in PPLO broth medium for 24 h and then stained with LIVE/DEAD BacLight, which made the live bacteria appear green and the dead bacteria red. Images of orthogonal sections (**A**) and topographical images (**B**) were observed using a confocal microscope. A representative image in three independent experiments is shown. Scale bar: 50 μm (**C** and **D**) *M. bovis* (strain M16) and *T. pyogenes* (strain T1) were co-cultured and stained with the LIVE/DEAD BacLight. Orthogonal sections (**C**) and topographical images (**D**) of biofilms were observed using a confocal microscope. A representative image in three independent experiments is shown. Scale bar: 50 μm. **E**–**J** Biofilms of single *M. bovis* (E: strain M16), *T. pyogenes* (**F**), and those co-cultured with these bacteria (**G**–**J**) were observed by SEM. Representative images are shown. **G** The red square was magnified in (**H**) to provide a better view. **H** Under the co-culture condition, *M. bovis* (yellow arrowheads) and *T. pyogenes* (red arrowheads) were connected. **I** Biofilm of *M. bovis* and *T. pyogenes* formed a large aggregation. The red square was magnified in (**J**) to provide a better view. **J** The boundary between the bacteria was obscured (green arrowheads). Scale bar of E–J: 5 μm. A representative image in three independent experiments is shown.

The biofilm of *M. bovis* (strain M16) was stained with LIVE/DEAD BacLight and observed using a confocal microscope (Figures 5A and B). A single culture of *M. bovis* showed aggregations of biofilm comprised of live bacteria*.* In contrast, the biofilm co-cultured with *M. bovis* and *T. pyogenes* was formed with both live and dead bacteria, with the surface of the biofilm especially covered in dead bacteria (Figures 5C and D).

The biofilm formation of single *M. bovis* (strain M16), *T. pyogenes* (strain T1), and the co-culture of these bacteria was analysed by SEM (Figures 5E–H). Aggregations of varying sizes were observed as *M. bovis* biofilms formed through adhesion between neighbouring bacteria (Figure 5E). In contrast, *T. pyogenes* showed no biofilm formation, and only a slight aggregation of bacterial cells was observed in a single culture (Figure 5F).

*M. bovis* and *T. pyogenes* adhered in co-culture, and no bacterium had cell wall damage or erosion (Figures 5G and H). Interestingly, some large aggregates of bacteria (> 40 μm), comprising *M. bovis* and *T. pyogenes*, were observed (Figure 5I). This aggregation was not observed in single cultures of *M. bovis* and *T. pyogenes*. The boundary between the aggregating bacteria was unclear (Figure 5J, indicated by green arrowheads).

## Discussion

*M. bovis* causes chronic pneumonia in calves, which is challenging to treat with antibiotics. It is widely recognised that bacteria in biofilms can become significantly more resistant to antimicrobial agents, with resistance ranging from 10 to 1000 times more compared to planktonic bacteria [20]. In a previous study, it was observed that the tracheal tissues of calves experimentally infected with *M. bovis* exhibited fimbriae distortion and oedema of the attached cells. Furthermore, in some cases, *M. bovis* was found to reside in both ciliated and non-ciliated epithelial cells [21]. However, biofilm formation of *M. bovis* in vivo has not been observed.

Consequently, we performed autopsies on calves that were naturally infected with chronic *Mycoplasma* pneumonia and evaluated the biofilm formation of *M. bovis*. Calf 1 and Calf 2 showed pathological characteristics, including caseous necrosis foci in the lungs. *T. pyogenes* is an important secondary pathogen and is most commonly isolated from lung lesions in BRDC [22]. Co-infection with *M. bovis* and *T. pyogenes* has also been identified as a risk factor for severe BRDC [22]. In this study, not only was *M. bovis* isolated as dominant bacteria from the trachea and lungs, but *T. pyogenes* was also isolated, suggesting that these pathogens were significant factors leading to lesions.

We conducted a morphological analysis of the trachea from calves with pneumonia*.* Compared to control calves, calves with *Mycoplasma* pneumonia had fewer cilia and clumping on the epithelial cells of the tracheal mucosa. Additionally, *M. bovis* antigen was detected in the epithelial cells of the trachea using a fluorescent microscope. A previous study showed that *Mycoplasma fermentans* infection caused the clumping of the cilia tips in mice [23]. Notably, ciliary activity is reduced as the infection progresses, and cilia form and shape become shorter [23].

It has been reported that the production of hydrogen peroxide and other reactive oxygen species (ROS) may be important factors in the pathogenicity of mycoplasmas, as they cause damage to cell membranes [24, 25]. Furthermore, the genes involved in producing hydrogen peroxide and ROS were encoded in the genome of commensal mycoplasma [26]. Interestingly, *M. bovis* produces hydrogen peroxide, which damages epithelial cells in vivo and in vitro [27, 28]. Exposure to high concentrations of hydrogen peroxide inhibited tracheal ciliary movement and induced apoptosis in epithelial cells [29, 30]. This damage to the respiratory mucosa reduces the immune barrier and triggers a local inflammatory response. Here, we suggest that *M. bovis* induces clumping and removes cilia from the trachea by producing hydrogen peroxide, which leads to a decline in tracheal immunity. In this study, bacterial aggregation structures were observed adhering to cilia from both calves with *Mycoplasma* pneumonia.

Reports have also indicated that large, mature biofilm structures, in which networks of bacteria are embedded within structured matrices, have been observed in the nasopharyngeal tissue of mice experimentally infected with *Streptococcus pneumoniae* (*S. pneumoniae*) [31]. This large biofilm structure of *S. pneumoniae* was associated with increased antibiotic resistance. In this study, we used SEM to observe bacterial structures on the cilia. These structures exhibited a pleomorphic or coccoid shape, measuring approximately 0.4–0.5 μm. Therefore, we suspected that *M. bovis* was the bacterial structure on the cilia and that aggregation structures were biofilms of *M. bovis* in vivo. This finding was further associated with antibiotic resistance in *Mycoplasma* pneumonia in calves. Notably, *M. bovis* was detected on the cilia via SEM, but it was not identified by fluorescence microscopy.

Tracheal tissues were prepared for SEM by fixing them with a solution containing half-strength Karnovsky’s and 1% osmium tetroxide. The osmium tetroxide in the solution fixes the phospholipids present in the biological membranes. *M. bovis* lacks a cell wall, so osmium fixation is useful for SEM observation [32]. We speculated that structures on the cilia were maintained in treatment for SEM but not in treatment for fluorescence microscopes. Additionally, *M. bovis* antigen was detected in the submucosal gland of *Mycoplasma* pneumonia Calf 2 by fluorescence microscope.

It has been reported that influenza viruses attach to and replicate in the tracheal submucosal glands [33]. Different subtypes of influenza viruses have different characteristics, and their adhesion to these glands is related to their pathogenicity. However, no reports have indicated the detection of *Mycoplasma* spp. in tracheal submucosal glands; therefore, further analysis is required to determine the detailed mechanism of *M. bovis* adherence to tracheal submucosal glands. *T. pyogenes* was also isolated from the trachea in calves with pneumonia, and it was thought to be involved in the biofilm formation of *M. bovis*. Thus, we examined biofilm formation during in vitro co-cultivation of *M. bovis* and *T. pyogenes*.

The methods for evaluating *M. bovis* biofilms were previously reported to be unstable [15], so we could not reproduce them directly. Consequently, we first identified the most suitable medium to assess the biofilm formation of *M. bovis.* We then determined that *M. bovis* cultured in a PPLO broth medium produced more biofilm than in other media. Additionally, we found that DMEM and RPMI promoted biofilm formation more than DMEM FBS and RPMI FBS, respectively. Moreover, a previous study evaluated *M. bovis* biofilm using the PPLO-rich growth medium [15]. However, in our study, *M. bovis* biofilm formation was lowest in the PPLO-rich medium.

In contrast, the PPLO broth medium, which did not have added growth nutrients such as horse serum and yeast extract, yielded the highest biofilm formation. Biofilm production generally increases in environments where bacterial growth is difficult [11]. One study reported that *Pseudomonas aeruginosa* (*P. aeruginosa*) promotes cell attachment and biofilm maturation in an environment lacking carbon, nitrogen, and phosphorus [34]. In *E. coli*, an SOS response occurs when amino acids are deficient, and biofilm formation is regulated [35]. For *M. bovis*, chaperone protein DnaK and elongation factor Tu are known to be involved in biofilm formation [36]. Since bovine serum is rich in nutritional factors essential for the growth of *M. bovis*, we speculated that adding serum to culture media may regulate adhesion to plates and suppress biofilm formation factors, resulting in reduced biofilm. Thus, our data suggested that a PPLO broth without added nutrients for growth was suitable for evaluating the biofilm formation of *M. bovis*.

We evaluated the biofilm formation abilities of seventeen species, including *M. bovis* in PPLO broth medium. Strains M1 and M7 were strong biofilm producers, while strains M5, M11, M12, and M14 were weak. Biofilm formation is characterised by three stages: attachment, maturation, and dispersion [11]. The attachment stage is a crucial initial step in biofilm formation. *P. aeruginosa* uses flagella for movement [37], and *S. aureus* adheres to host-derived EPS for attachment [38]. Previous studies have indicated that the variable surface proteins B and O type, which are among the adhesion factors expressed by the wild strain *M. bovis*, form prolific biofilms [15]. The difference in biofilm production ability among *M. bovis* strains was thought to be due to the expression levels of various adhesion factors. In this study, *M. bovis* was observed, using a confocal microscope, as aggregations of varying sizes and scattered formations consisting of live bacteria. Additionally, a moniliform structure bound together by filamentous material was observed using an electron microscope.

Previous research has demonstrated that a thick filamentous matrix connects bacterial cells in *Clostridium difficile* (*C. difficile*) and *S. aureus* biofilms [39, 40]. This matrix was thought to be an extracellular matrix (ECM) released by bacteria [40]. The filamentous matrix in biofilms connects neighbouring groups of bacteria, which serves as an initial step in aggregation [39, 40]. Furthermore, nuclease treatment inhibited *Mycoplasma hyopneumoniae* (*M. hyopneumoniae*) biofilm formation, thus suggesting that extracellular DNA released outside the cell is a crucial step for biofilm formation [41]. Although it has been reported that the *M. bovis* biofilm consists of viable bacteria, a detailed morphological analysis has yet to be conducted [15]. Here, we observed aggregations of bacteria and moniliform structures between *M. bovis* cells. This finding suggests that *M. bovis* also releases ECM or DNA to connect with neighbouring bacteria. Subsequently, *M. bovis* then forms compact microcolonies and biofilm.

*M. bovis* and *T. pyogenes* are pathogens that cause pneumonia in cattle. It is not uncommon for both bacteria to be detected from the same lesion, as in this study [10, 42]. The expression levels of multiple virulence genes in *T. pyogenes* have been found to increase upon co-infection with *E. coli* and *F. necrophorum* in mice [9]. Additionally, it is assumed that co-infection affects the formation of biofilms in pathogens, and understanding this process is crucial to comprehend the pathology of pneumonia. Thus, we examined the polymicrobial relationship between *M. bovis* and *T. pyogenes* during biofilm formation. The biofilm formation of *M. bovis* (PG45 and strain M16) was synergistically increased when co-cultured with 1 × 10^4^, 1 × 10^5^, and 1 × 10^6^ CFU/well of *T. pyogenes* (strain T1 and T2). In contrast, the biofilm formation with 1 × 10^7^ CFU/well of *T. pyogenes* was lower than with 1 × 10^5^ and 1 × 10^6^ CFU/well of* T. pyogenes.*

The biofilm resulting from co-cultivation with *M. bovis* (strain M16) and *T. pyogenes* comprised both live and dead bacteria, with a higher proportion of dead bacteria than single cultures. *S. aureus*-induced apoptosis in *C. albicans* was characterised by features such as intracytoplasmic disorganisation, cell membrane discontinuity, vacuole formation, and chromatin condensation [43]. The induction of apoptosis was not a result of cell-to-cell contact but rather of the presence of the *S. aureus* supernatant*.* Our study suggests that a co-cultivation condition induced apoptosis in *M. bovis*, *T. pyogenes*, or both bacteria, which was notably observed when the number of *T. pyogenes* was high. However, SEM observed adherence of *M. bovis* (strain M16) and *T. pyogenes* to each other and the formation of large bacterial aggregates. *S. aureus* caused the death of *Aspergillus fumigatus*, but in co-cultivation, the morphological characteristics of the biofilm produced by these bacteria were similar to those observed in vivo [40].

Furthermore, eDNA acted as a crucial EPS component shared by *S. aureus* and *P. aeruginosa* in co-culture biofilms, facilitating interspecies interactions by promoting the formation of compact microcolony structures during biofilm development [13].

This cell-to-cell communication is a major coordination factor in biofilm formation [44, 45]. A previous study indicated that *M. bovis* has a very limited genome but can produce biofilms that help it survive environmental stress, such as exposure to disinfection [15]. Our study demonstrates that combining *M. bovis* and *T. pyogenes* can form a large microcolony. This finding suggests that *M. bovis* collaborates with other bacteria to form a mature biofilm, increasing resistance to environmental disinfections, host immunity, and antimicrobials.

While the morphological traits of polymicrobial colonisation were somewhat similar to those seen in the trachea of calves with *Mycoplasma* pneumonia in vivo, they were not entirely identical compared to single cultures in vitro. Previous reports indicate that the biofilm grown on the epithelial cells exhibits phenotypes similar to those observed during in vivo colonisation [31]. Epithelial cells play a crucial role in the biofilm formation process by potentially facilitating optimal interbacterial signalling and the expression of colonisation-associated factors necessary for biofilm formation through adherence to in vivo ligands. Our study, however, is the first to expound on the interactions between *M. bovis* and *T. pyogenes* during biofilm formation. Moreover, these mechanisms may be involved in the progression of pathology in *Mycoplasma* pneumonia. Further analysis using bovine epithelial cells is required to elucidate the mechanism of biofilm formation of *M. bovis*.

In conclusion, we observed mature biofilms of *M. bovis* on the tracheae of calves naturally infected with pneumonia and established a method for evaluating the biofilm formation of *M. bovis* in vitro. *M. bovis* biofilms were observed as aggregations of various sizes and filamentous matrices connecting neighbouring groups, suggesting that *M. bovis* releases ECM or DNA to connect to neighbouring bacteria and subsequently form compact microcolonies. Additionally, co-cultivation with *M. bovis* and *T. pyogenes* caused significant biofilm formation. This study indicated that the interaction between *M. bovis* and *T. pyogenes* led to increased resistance to antimicrobial agents, thereby exacerbating the progression of chronic *Mycoplasma* pneumonia.

## Supplementary Information

**Additional file 1. Morphological analysis of trachea tissues isolated from control and* Mycoplasma* pneumonia in calves. **Trachea tissues from no clinical respiratory symptoms control and *Mycoplasma *pneumonia in calves were stained with haematoxylin and eosin. Cilia were detected on the epithelial cells of control calves. Scale bar: 32 μm.Trachea tissues from control and *Mycoplasma *pneumonia in calves were stained with cytokeratin-18 antibody, *M. bovis* antibody, and DAPI solution and analysed by fluorescence microscope. Cytokeratin-18 is stained in red, *M. bovis* is stained in green, and the nucleus is stained in blue. These images are overlayed. Representative images are shown. Positive areas of *M. bovis* were detected in epithelial cells of trachea tissues from *Mycoplasma *pneumonia in calf. Scale bar: 100μm.Trachea mucosa from control and *Mycoplasma *pneumonia in calves were analysed by SEM. Scale bar: 10μm.The surface of the tracheal mucosa from the control calf was covered all over with cilia.Bacterium-like aggregation structures on the tracheal mucosa from *Mycoplasma *pneumonia in calves were observed. The red square was magnified into show better.Bacteria were detected on the bacterium-like aggregation structures.

**Additional file 2. Morphological analysis of* Mycoplasma bovis* by scanning electron microscope. **Biofilm formation of *Mycoplasma bovis* PG45and strain M2were analysed by scanning electron microscope. Bacterium aggregation structures were observed. Scale bar: 10 μm.

## Data Availability

All data analyzed in this study are included in this article and its supplementary files.
