# Supplementary figures and images for: Biofilm characterisation of Mycoplasma bovis co-cultured with Trueperella pyogenes
Source: Vet Res. 2025 Jan 30;56:22. doi: 10.1186/s13567-025-01468-1 (PMC11783866; doi:10.1186/s13567-025-01468-1)

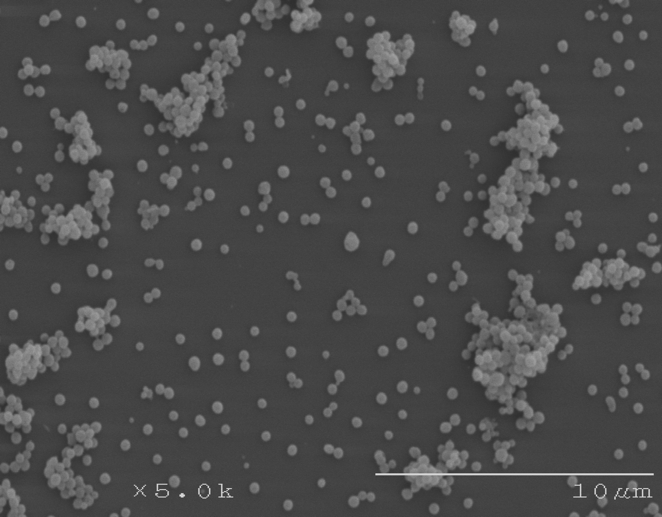

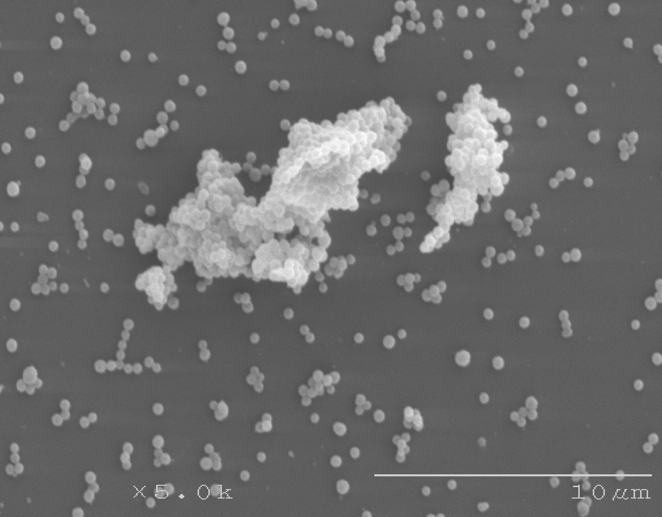


B

A

Supplement: Supplementary file 2 — Additional file 2. Morphological analysis of Mycoplasma bovis by scanning electron microscope. Biofilm formation of Mycoplasma bovis PG45and strain M2were analysed by scanning electron microscope. Bacterium aggregation structures were observed. Scale bar: 10 μm. [file 13567_2025_1468_MOESM2_ESM.docx]
