# Supplementary material for: Biofilm characterisation of Mycoplasma bovis co-cultured with Trueperella pyogenes
Source: Vet Res. 2025 Jan 30;56:22. doi: 10.1186/s13567-025-01468-1 (PMC11783866; doi:10.1186/s13567-025-01468-1)
Supplement: Supplementary file 2 [file 13567_2025_1468_MOESM1_ESM.docx]

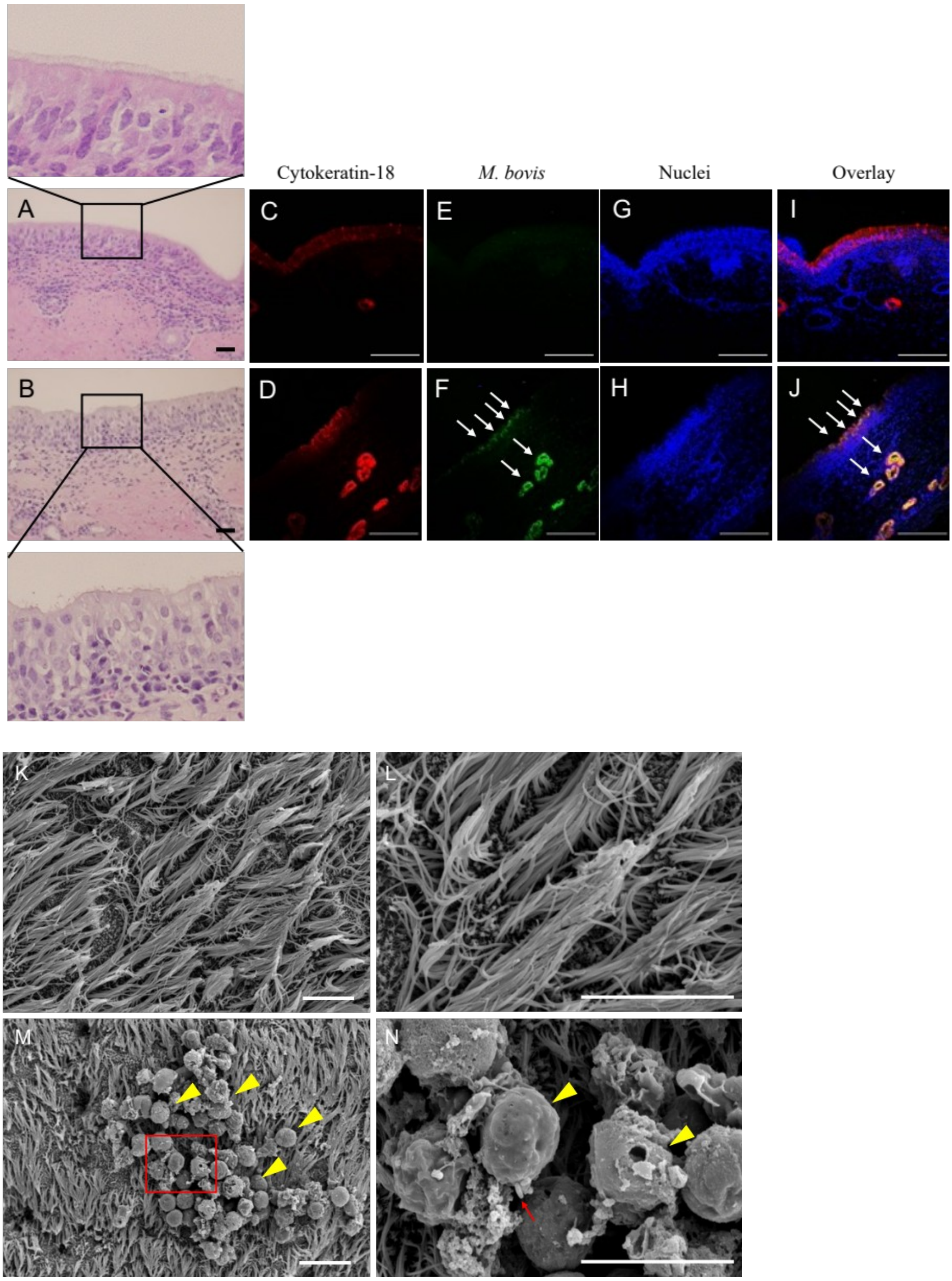

Cytokeratin-18
M. bovis
Nuclei
Overlay
A
B
C
D
E
F
G
H
I
J
K
L
M
N
